# Association of Pelvic Trauma With Rates of Cesarean Section, Sexual Dysfunction, and Genitourinary Dysfunction in a National Database

**DOI:** 10.5435/JAAOSGlobal-D-22-00166

**Published:** 2023-04-10

**Authors:** Kevin Chen, Sarah Bhattacharjee, Henry Seidel, Daryl B. Dillman, Jason A. Strelzow

**Affiliations:** From the Pritzker School of Medicine at the University of Chicago, Chicago, IL (Mr. Chen, Dr. Bhattacharjee, and Mr. Seidel); and the Department of Orthopaedic Surgery and Rehabilitation Medicine, University of Chicago Medicine, Chicago, IL (Dr. Dillman and Dr. Strelzow).

## Abstract

Pelvic fractures are severe injuries that can drastically affect a woman's quality of life through sexual dysfunction (SD), genitourinary dysfunction (GD), and increasing the potential need for future cesarean section (C-section). Limited research has captured long-term outcomes after pelvic fractures in women of childbearing age. This study aimed to determine the association between pelvic fractures and rates of C-section, SD, and GD.

**Methods:**

All women of childbearing age who sustained a pelvic fracture were identified in a national insurance database. A comparison group of patients with lower extremity long-bone fractures was selected. Patients who gave birth after injury were additionally identified. A minimum of 5 years of follow-up was required for inclusion. Rates of C-section, SD, and GD were compared between cohorts. Multivariate logistic regression analysis was conducted with the inclusion of diabetes, tobacco, hypertension, obesity, and advanced maternal age.

**Results:**

A total of 6,174 patients with pelvic fracture and 27,154 control fracture patients were identified. 434 patients with pelvic fracture (7.0%) and 1,258 control fracture patients (4.6%) gave birth after fracture. Patients with pelvic fracture had a significantly higher rate of C-section (50.0% versus 38.8%, *P* < 0.001), SD diagnosis (10.9% versus 8.8%, *P* < 0.001), and urinary retention diagnosis (3.5% versus 2.8%, *P* < 0.001). No significant difference in global GD diagnosis was identified. Multivariate analyses showed that pelvic fracture was associated with C-section (odds ratio [OR]: 1.78; 95% confidence interval [95% CI]: 1.42 to 2.23, *P* < 0.001), SD diagnosis (OR: 1.23; 95% CI: 1.12 to 1.35, *P* < 0.001), and urinary retention diagnosis (OR: 1.35; 95% CI: 1.15 to 1.57, *P* < 0.001).

**Discussion:**

Pelvic fractures confer an intrinsic level of risk of C-section, SD, and urinary retention that is elevated beyond what would be expected from a traumatic lower extremity injury alone. Treating orthopaedic surgeons should actively counsel women regarding increased risks, openly discuss postinjury sequelae, and coordinate interspecialty care beyond initial treatment of acute trauma.

Pelvic fractures are severe injuries that often result from high-energy blunt trauma and comprise approximately 10% of all blunt trauma admissions.^1^ With advances in their orthopaedic management decreasing patient mortality, more attention has been directed toward the long-term outcomes of these injuries. This is particularly important for women when considering their greater susceptibility to pelvic fracture and the potential effects of such trauma on pregnancy and childbirth.^2,3^ The high frequency of concomitant intrapelvic injuries elicits concerns regarding the ramification of pelvic fracture on future reproductive, sexual, and genitourinary functions.^1,2,3,4^

A central question for women wishing to have children after pelvic fracture is, whether vaginal delivery is possible? It has been postulated that residual deformities of the pelvis and retained surgical metalwork can limit the widening of the pelvic outlet necessary to accommodate childbirth, which can encourage or require patients to pursue a cesarean section (C-section).^3,4^ This would seemingly be supported by the literature on the topic, with women with prior pelvic fracture reported to experience higher rates of C-section.^3,4,5,6,7,8^ These studies are limited by small sample size, investigation at single institutions, and not controlling for other C-section risk factors, such as a history of C-section. Because 92% of women with prior C-section undergo repeat C-section in future deliveries, it is unclear whether elevated C-section rates after pelvic fracture are due to the injury itself or prior C-section.^9^ Furthermore, control groups were often absent, leaving pelvic fracture comparisons relative to population norms, and patients often had widely varying follow-up times, leaving unaccounted those who gave birth further out from injury.

Similarly, pelvic fractures have been associated with persistent symptoms of sexual dysfunction (SD)^4,10-12^ and genitourinary dysfunction (GD),^4,7^ which likely arise from concomitant injury to nearby soft-tissue and key nervous, reproductive, and urinary structures. However, because many of these studies assess dysfunction with questionnaires instead of confirmed diagnosis, reliance on data from patient self-reporting and nonvalidated questionnaires may cause survey and measurement bias. Moreover, few studies have assessed SD/GD outcomes specifically in women.

Often involved in the short-term and long-term care of women with pelvic fractures, orthopaedic surgeons can serve as a valuable resource for these patients by helping guide the expectations of reproductive, sexual, and genitourinary functions in the years after injury. However, the lack of large population-based data hampers optimal counseling of patients. We aimed to study the association between pelvic fracture and postinjury rates of C-section, SD diagnosis, and GD diagnosis in women of childbearing age using a large national database. We hypothesized that patients with pelvic fracture would have higher postinjury rates of C-section, SD, and GD compared with a control cohort with injuries of similar effect energy but spare the pelvis.

## Methods

### Database

This retrospective study was conducted using the PearlDiver national insurance claims database (PearlDiver Inc). Comprising 51 million records of a deidentified orthopaedic patient population from 2005 to 2014, the PearlDiver database is searchable using the International Classification of Diseases (Ninth Revision, ICD-9; 10th Revision, ICD-10) codes and Current Procedural Terminology codes. Because all records were anonymous and compliant with HIPAA standards, this study was determined to be exempt from the Institutional Review Board under IRB18-0215.

### Patient Selection

**Figure 1:**
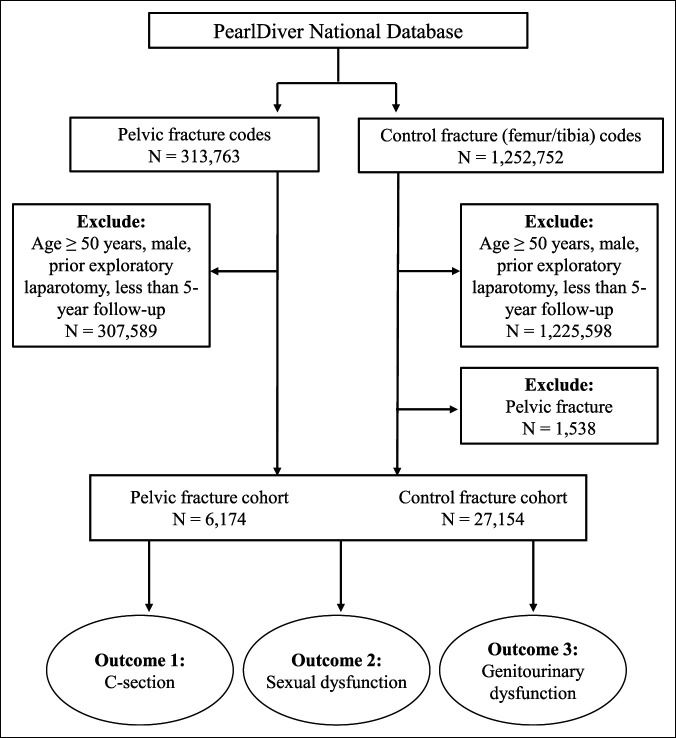
Flow chart depicting the inclusion and exclusion criteria for creating the pelvic and control fracture cohorts used to assess our primary outcome of cesarean section and secondary outcomes of sexual dysfunction and genitourinary dysfunction.

We created a pelvic fracture cohort by identifying all patients in the database with a recorded ICD-9/10 diagnostic code for pelvic fracture. Within this cohort, patients were excluded if they were male, were considered outside of childbearing age (50 years or older), had less than 5 years of active database records after injury, and had a history of exploratory laparotomy preinjury. We similarly created a control fracture cohort by identifying all patients with ICD codes for fractures to the shaft of the femur or tibia and subsequently excluding those who did not meet the same set of the abovementioned criteria. The femur and tibia were chosen as controls given that the pelvis and lower extremity long bones require similar levels of traumatic energy to fracture and similarly, are frequently treated surgically. Any patient in the control group with a pelvic fracture before or after long-bone fracture was excluded. Rates of C-section, SD, and GD were compared between the pelvic and control fracture cohorts (Figure 1).

To assess our primary outcome of C-section, patients from both pelvic and control fracture cohorts who gave birth within 5 years after injury were identified by Current Procedural Terminology codes for either C-section or vaginal delivery. Because past C-section is a risk factor of future C-sections, we excluded births by patients who had a previous C-section.^9^ The C-section rate was calculated as the number of C-sections of the total number of births. Finally, the Elixhauser Comorbidity Index and the comorbidities of diabetes, tobacco, hypertension, obesity, and advanced maternal age were evaluated for differences between groups and included as independent predictors in statistical analyses to control for their effect on outcomes. Advanced maternal age was defined as 35 years or older.^13^

A secondary analysis of SD/GD rates and comorbidities within the pelvic and control groups were compared at 5-year follow-up. The SD/GD rate was calculated as the number of patients with at least one SD/GD diagnosis of the total number of patients sustaining a fracture. Because we were interested in capturing any type of SD after injury, we broadly defined SD based on diagnostic codes listed in the International Classification of Diseases: Female Sexual and Pelvic Dysfunction.^14^ SD diagnoses included low libido; dyspareunia; vaginismus; sexual aversion; orgasmic disorder; and pain in the vaginal, perineal, or pelvic regions. GD outcome was defined as common genitourinary diagnoses that have been linked to pelvic fractures, including nocturia, stress incontinence, frequency, and retention.^4,7,15^ A subanalysis assessing the association between each GD diagnosis individually and pelvic fracture was also conducted. A full list of procedure and diagnoses codes is presented in Supplemental Table 1, http://links.lww.com/JG9/A276.

### Statistical Analysis

Univariate logistic regressions were conducted to determine the association of pelvic fracture and comorbidities with the outcomes of interest. The alpha value was set at 0.05. For each outcome, the variables notable in the univariate were included in a subsequent multivariate logistic regression. Adjusted odds ratios were calculated for each variable with an alpha value of 0.05. Regression analyses were calculated using R statistical package.

## Results

A total of 313,763 patients with pelvic fracture and 1,252,752 control fracture patients were identified in the database. After a review of exclusion criteria, a final population of 6,174 patients with pelvic fracture (2.0%) and 27,154 control fracture patients (2.2%) was analyzed.

### Cesarean Section

**Figure 2:**
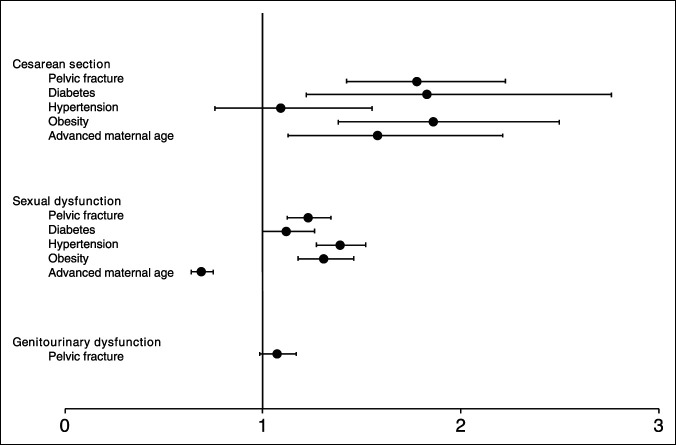
Forest plot showing the OR of risk factors of cesarean section (adjusted OR for pelvic fracture: 1.78, 95% confidence interval [95% CI]: 1.42 to 2.23, *P* < 0.001), sexual dysfunction (adjusted OR for pelvic fracture: 1.23, 95% CI: 1.12 to 1.35, *P* < 0.001), and genitourinary dysfunction (unadjusted OR for pelvic fracture: 1.07, 95% CI: 0.98 to 1.17, *P* = 0.11). OR = odds ratio

At the 5-year follow-up, 434 patients (7.1%) in the pelvic fracture cohort and 1,258 patients (4.6%) in the control fracture cohort had 444 deliveries and 1,267 deliveries, respectively (Table 1). Patients with pelvic fracture demonstrated a significantly higher rate of C-section compared with control subjects (50.0% versus 38.8%, *P* < 0.001). Univariate and multivariate analyses continued to demonstrate a significantly higher rate of C-section in the pelvic fracture cohort (univariate odds ratio [OR]: 1.63, 95% confidence interval [95% CI] 1.31 to 2.03, *P* < 0.001; multivariate OR: 1.78, 95% CI 1.42 to 2.23, *P* < 0.001) (Table 2; Figure 2). Diabetes, obesity, and advanced maternal age were also significant in the multivariate analysis (diabetes OR: 1.83, 95% CI 1.22 to 2.76, *P* = 0.003; obesity OR: 1.86, 95% CI 1.38 to 2.50, *P* < 0.001; advanced maternal age OR: 1.58, 95% CI 1.13–2.21, *P* = 0.008).

**Table 1:** Demographics of Pelvic and Control Fracture Patients With Births After Injury

	All Fracture (n = 1,692)	Pelvic Fracture (n = 434)	Control Fracture (n = 1,258)	*P* value
Deliveries, n (%)	1,711 (100.0)	444 (100.0)	1,267 (100.0)	
Cesarean delivery,^a^ n (%)	714 (41.7)	222 (50.0)	492 (38.8)	<0.001
Vaginal delivery,^a^ n (%)	997 (58.3)	222 (50.0)	775 (61.2)	<0.001
Comorbidities, n (%)				
Diabetes	115 (6.8)	21 (4.8)	94 (7.5)	0.062
Tobacco	363 (21.5)	90 (20.7)	273 (21.7)	0.673
Hypertension	161 (9.5)	30 (6.9)	131 (10.4)	0.032
Obesity	230 (13.6)	44 (10.1)	186 (14.8)	0.015
Advanced maternal age^b^	159 (9.4)	21 (4.8)	138 (11.0)	<0.001
Elixhauser Comorbidity Index	1.34 ± 1.72	1.28 ± 1.69	1.36 ± 1.72	0.402

a Deliveries within five years of injury.

b Advanced maternal age is defined as 35 years and older.

**Table 2:** Multivariate Logistic Regression of Risk Factors of Cesarean Section

Variables	OR (95% CI)	*P* value
Pelvic fracture	1.78 (1.42-2.23)	<0.001
Diabetes	1.83 (1.22-2.76)	0.003
Hypertension	1.09 (0.76-1.55)	0.638
Obesity	1.86 (1.38-2.50)	<0.001
Advanced maternal age^a^	1.58 (1.13-2.21)	0.008

OR = odds ratio, CI= confidence interval

a Advanced maternal age is defined as 35 years and older.

#### Sexual Dysfunction

At the 5-year follow-up, patients with pelvic fracture were significantly more likely to be diagnosed with SD compared with control subjects (10.9% versus 8.8%, *P* < 0.001) (Supplemental Table 2, http://links.lww.com/JG9/A276). Univariate and multivariate analyses continued to demonstrate a significantly higher rate of SD diagnosis in the pelvic fracture cohort (univariate OR: 1.27, 95% CI 1.16 to 1.39, *P* < 0.001; multivariate OR: 1.23, 95% CI 1.12 to 1.35, *P* < 0.001) (Table 3). Tobacco, obesity, and advanced maternal age were also independently associated with postinjury SD diagnosis in the multivariate analysis (tobacco OR: 1.39, 95% CI 1.27 to 1.52, *P* < 0.001; obesity OR: 1.31, 95% CI 1.18 to 1.46; *P* < 0.001; advanced maternal age OR: 0.69, 95% CI 0.64 to 0.75, *P* < 0.001).

**Table 3:** Multivariate Logistic Regression of Risk Factors of Sexual Dysfunction

Variables	OR (95% CI)	*P* value
Pelvic fracture	1.23 (1.12-1.35)	<0.001
Diabetes	1.12 (1.00-1.26)	0.056
Tobacco	1.39 (1.27-1.52)	<0.001
Obesity	1.31 (1.18-1.46)	<0.001
Advanced maternal age^a^	0.69 (0.64-0.75)	<0.001

OR = odds ratio, CI = confidence interval

a Advanced maternal age is defined as 35 years and older.

#### Genitourinary Dysfunction

At the 5-year follow-up, no significant difference was observed in GD diagnosis rates between patients with pelvic fracture and control subjects (11.9% versus 11.2%, *P* = 0.105) (Supplemental Table 2, http://links.lww.com/JG9/A276). Because no significant association was observed between pelvic fracture and GD diagnosis in the univariate analysis, no multivariate analysis was conducted.

In the subanalysis that disaggregated the broad category of GD into individual diagnoses of nocturia, frequency, stress incontinence, and retention, patients with pelvic fracture were significantly more likely to be diagnosed with retention compared with control subjects (3.5% versus 2.8%, *P* < 0.001). Univariate and multivariate analyses continued to demonstrate a significantly higher rate of retention diagnosis in the pelvic fracture cohort (univariate OR: 1.25, 95% CI 1.07 to 1.45, *P* = 0.005; multivariate OR: 1.35, 95% CI 1.15 to 1.57, *P* < 0.001) (Table 4). Diabetes, tobacco, hypertension, and advanced maternal age were also independently associated with postinjury retention diagnosis in the multivariate analysis (diabetes OR: 1.83, 95% CI 1.55 to 2.16, *P* < 0.001; tobacco OR: 1.47, 95% CI 1.27 to 1.70, *P* < 0.001; hypertension OR: 1.60, 95% CI 1.37 to 1.87, *P* < 0.001; advanced maternal age OR: 1.25, 95% CI 1.09 to 1.45, *P* = 0.002). Because no significant association was observed between pelvic fracture and nocturia, urinary frequency, or stress incontinence in the univariate analysis, no multivariate analysis was conducted.

**Table 4:** Multivariate Logistic Regression of Risk Factors of Urinary Retention Subanalysis

Variables	OR (95% CI)	*P* value
Pelvic fracture	1.35 (1.15-1.57)	<0.001
Diabetes	1.83 (1.55-2.16)	<0.001
Tobacco	1.47 (1.27-1.70)	<0.001
Hypertension	1.60 (1.37-1.87)	<0.001
Obesity	1.10 (0.92-1.30)	0.280
Advanced maternal age^a^	1.25 (1.09-1.45)	0.002

OR = odds ratio, CI = confidence interval

a Advanced maternal age is defined as 35 years and older.

## Discussion

Women who sustain pelvic fractures often face pressing questions regarding their long-term reproductive, sexual, and genitourinary functions. Prior studies have suggested increased rates of C-section, SD, and GD after pelvic fractures.^3,4,5,6,7,8,10-12^ However, these studies have key limitations, such as small sample size and reliance on questionnaires. A clearer picture of the long-term effect of pelvic fracture on these outcomes is crucial and may allow orthopaedic surgeons to better counsel patients with these injuries. In this study, we found a markedly higher rate of C-section, SD diagnosis, and retention diagnosis in women after pelvic fracture compared with control subjects.

### Cesarean Section

Past studies reported that C-section rates after pelvic fracture range from 9% to 89%, with this variability likely because of sample sizes of less than 30 patients for all but one study.^3,4,6,7,16^ One systematic review identified a postinjury C-section rate of 42% after analysis of 137 C-section–naïve patients.^5^ Because prior C-section promotes future C-sections, we similarly excluded births after prior C-section to account for its large confounding effect and found a 50% C-section rate in a pelvic fracture cohort of 434 patients.^9^ With a similar rate as prior studies and the greater overall generalizability of findings from a national database, our results suggest that the C-section rate after pelvic fracture may be much higher than the general population. The C-section rate for women without prior C-section was reported to be 23% by the Centers for Disease Control and Prevention, which is much lower than the 50% C-section rate identified in our pelvic fracture cohort.^9^ The C-section rate in our control cohort is also higher than the national average, paralleling findings from a past study with similar comparison groups.^4^ Considering that patients in the control cohort sustained injury severe enough to fracture the femur or tibia, obstetricians may have chosen C-section for these patients because of concern about injury proximity complicating vaginal delivery.

Because many studies compared pelvic fracture cohorts with population norms, we specifically used a control group to better distinguish between the effect of pelvic fracture itself and that of overall trauma on C-section risk. The high level of significance for pelvic fracture after controlling for comorbidities in multivariate analyses validates our hypothesis and suggests that pelvic fracture is predictive of future C-sections. Specifically, patients with pelvic fracture can expect an almost doubled odds of C-section compared with those with injuries that possess similar effect energy but spare the pelvis. Proper counseling of these women regarding their increased risk is essential because C-section poses notable risk to both mother and fetus with its 15% complication rate and long-term implications for future pregnancies related to primary C-section.^17^

There remains some controversy over whether elevated C-section rates are driven by patient and surgeon preference or by true anatomical indication. With 77% of obstetricians reporting no experience treating patients with pelvic fracture and only 23% thinking that C-sections are not always indicated for this patient population, physician bias and resulting recommendation can augment preexisting patient preference for C-section.^4,7^ Many studies, however, do not suggest that pelvic fractures preclude the ability to deliver vaginally and instead find that successful vaginal delivery is possible even with prior surgical treatment and retained pelvic instrumentation.^3,7^ Conversely, anatomical indications, such as residual deformities or retained instrumentation, may be driving elevated C-section rates. If there is clinical suspicion that pelvic outlet widening is not possible, then it is imperative for obstetricians to be aware of prior pelvic fracture.^4,7^ Clearly, additional work is needed to fully understand these underlying factors and determine whether a scheduled elective C-section might be indicated, which could lessen the likelihood of failed vaginal delivery and emergency C-section.

### Sexual Dysfunction

We observed a much lower rate of SD diagnosis after pelvic fracture (12%) than 25% to 62% previously reported.^4,10-12^ This difference is likely due to the calculating rate based on questionnaires versus clinical diagnosis. Although questionnaires can better capture patient experience and allow patients to more freely disclose personal topics, they are ultimately subjective and prone to bias. Reliance on patients to recount their level of dysfunction preinjury and postinjury several years later can inflate the SD rate because of recall bias and confounding effect of older age at the time of response. The nonvalidated nature of these questionnaires can also lead to measurement bias and uncertainty regarding external validity. However, because SD is broadly underdiagnosed, we recognize that our use of diagnostic codes likely represents the lower bounds of the true rate.^18,19^ While patients with subclinical symptoms are more likely to report SD when directly asked in questionnaires, these same patients are unlikely to be included in our rate because they would not have met diagnostic criteria. Owing to the sensitive nature of sexual health, patients are also unlikely to bring up SD concerns unless prompted.^19,20^ Only 3% of women report SD without direct inquiry, compared with 19% on direct inquiry.^20^ Moreover, because physicians are unlikely to ask about SD, these conversations often may not occur at all.^18,19^ These factors may contribute to why we observed advanced maternal age to be associated with lower likelihood of SD diagnosis. Rather than being less likely to experience postinjury SD, older women may be facing a greater degree of SD underdiagnosis than younger women. In any case, our results suggest a sizeable discrepancy between what patients with pelvic fracture are experiencing regarding SD within the home setting and what is getting diagnosed within the clinical setting.

As hypothesized, we observed a markedly higher rate of SD diagnosis for patients with pelvic fracture relative to control subjects.^10-12^ Because our definition of SD was more inclusive than past studies defining SD as solely dyspareunia, these findings can help clinicians manage patient expectations of overall sexual functioning postinjury. Finally, SD is higher in women and a common occurrence after nonpelvic trauma, such as lower extremity long-bone fractures.^21^ Thus, the way we constructed our control group of femur and tibial fractures may have inflated the rate of SD within this cohort relative to population norms. The high level of significance we observed despite this inflation suggests that the odds of postinjury SD are higher than what our data indicate.

### Genitourinary Dysfunction

Past questionnaire-based studies reported that 21% to 49% patients reported at least one symptom of GD after pelvic fracture.^4,7^ We observed a much lower rate of GD after pelvic fracture at 12%, likely for the same reasons outlined earlier for SD. The lack of association between pelvic fracture and postinjury GD diagnosis was not in line with our hypothesis. Because a past study found markedly higher rates of GD in patients with more severe injury profiles, this observation could be explained by our inability to stratify patients by fracture severity.^4^

However, it is possible that pelvic fracture does not confer an increased GD risk in women. Compared with men, women more rarely sustain urethral injury after pelvic fracture because of the relative protection afforded by a short, internally located urethra.^22^ Consequently, women are largely insulated from postinjury onset of certain types of GD, and these diagnoses often only occur secondary to fractures severe enough to disrupt the pubic symphysis.^23^ Additional anatomical considerations could explain this observation. During bladder filling, physiological urethral compression against the anterior vaginal wall may attenuate the deleterious effects of mild concomitant injuries on continence.^24^ Thus, women could be more shielded from GD subtypes that involve increased or uncontrollable urination, such as nocturia, incontinence, and frequency.

Similar anatomical relationships could contribute to markedly higher rates of retention diagnosis after injury. We theorize that pelvic trauma leads to both pelvic floor muscle weakness and urethral stricture secondary to fibrotic scarring, which together inhibit bladder emptying.^2,24^ Because we did not stratify our cohorts by injury severity, we recognize that severe pelvic fractures may increase risk of overall GD or other GD subtypes that we could not detect.

Our study has a number of limitations. In using a national database, we did not have access to radiographs or charts to distinguish between various fracture types, injury severities, residual deformities, and fixation methods. Considering the heterogeneity of initial fracture severity and subsequent outcomes (eg, surgical versus nonsurgical management, retained instrumentation), combining these patients could have diluted our calculated statistical values. Furthermore, we could not examine these factors as independent predictors for our outcomes. The use of a database also necessitated reliance on accurate billing documentation. Finally, although this study demonstrates an elevated risk of C-section, SD, and retention, we cannot narrow down the cause and effect of pelvic trauma on these outcomes but rather note the correlation.

In conclusion, we found that pelvic fractures confer an intrinsic level of risk for C-section that is elevated beyond what would be expected from traumatic injury alone. Additionally associated with increased risk of subsequent SD and urinary retention diagnosis, pelvic fracture should be recognized by orthopaedic surgeons as a major risk factor of long-term reproductive, sexual, and genitourinary outcomes. With only half the patients with pelvic fracture being advised on future birth options and physician underestimation of SD prevalence, there is much that can be done to improve care for these women.^4,19^ We hope that our study can not only inform clinicians of the effect of pelvic fracture on these long-term outcomes but also encourage active counseling and coordinated interspecialty care for these women beyond their initial treatment of acute trauma.

## Supplementary Material

SUPPLEMENTARY MATERIAL

## References

[1] Demetriades D Karaiskakis M Toutouzas K Alo K Velmahos G Chan L: Pelvic fractures: Epidemiology and predictors of associated abdominal injuries and outcomes. J Am Coll Surgeons 2002;195:1-10. 10.1016/s1072-7515(02)01197-3 12113532

[2] Flint L Cryer HG: Pelvic fracture: The last 50 years. J Trauma Inj Infect Crit Care 2010;69:483-488. 10.1097/TA.0b013e3181ef9ce1 20838117

[3] Vallier HA Cureton BA Schubeck D: Pregnancy outcomes after pelvic ring injury. J Orthop Trauma 2012;26:302-307. 22048182 10.1097/BOT.0b013e31822428c5

[4] Copeland CE Bosse MJ McCarthy* ML : Effect of trauma and pelvic fracture on female genitourinary, sexual, and reproductive function. J Orthop Trauma 1997;11:73-81. 9057139 10.1097/00005131-199702000-00001

[5] Riehl JT: Caesarean section rates following pelvic fracture: A systematic review. Injury 2014;45:1516-1521. 24830904 10.1016/j.injury.2014.03.018

[6] Madsen LV Jensen J Christensen ST: Parturition and pelvic fracture. Follow-up of 34 obstetric patients with a history of pelvic fracture. Acta Obstet Gynecol Scand 1983;62:617-620. 6670466 10.3109/00016348309156259

[7] Cannada LK Barr J: Pelvic fractures in women of childbearing age. Clin Orthop Relat Res 2010;468:1781-1789. 20333494 10.1007/s11999-010-1289-5 PMC2881988

[8] Speer DP Peltier LF: Pelvic fractures and pregnancy. J Trauma Inj Infect Crit Care 1972;12:474-480. 10.1097/00005373-197206000-00004 5033493

[9] Martin JA Hamilton BE Sutton PD : Births: Final data for 2007. Natl Vital Stat Rep 2010;58:1-85. 21254725

[10] Walton AB Leinwand GZ Raheem O Hellstrom WJG Brandes SB Benson CR: Female sexual dysfunction after pelvic fracture: A comprehensive review of the literature. J Sex Med 2021;18:467-473. 33593705 10.1016/j.jsxm.2020.12.014

[11] Vallier HA Cureton BA Schubeck D: Pelvic ring injury is associated with sexual dysfunction in women. J Orthop Trauma 2012;26:308-313. 22011632 10.1097/BOT.0b013e31821d700e

[12] Rovere G Perna A Meccariello L : Epidemiology and etiology of male and female sexual dysfunctions related to pelvic ring injuries: A systematic review. Int Orthop 2021;45:2687-2697. 34378143 10.1007/s00264-021-05153-8 PMC8514382

[13] Adashek JA Peaceman AM Lopez-Zeno JA Minogue JP Socol ML: Factors contributing to the increased cesarean birth rate in older parturient women. Am J Obstet Gynecol 1993;169:936-940. 8238152 10.1016/0002-9378(93)90030-m

[14] Kingsberg SA Schaffir J Faught BM : Female sexual health: Barriers to optimal outcomes and a roadmap for improved patient-clinician communications. J Women's Health 2019;28:432-443. 10.1089/jwh.2018.7352 PMC6482896 30714849

[15] Kurian JJ Sen S Kishore R Srinivas R: Urethro vaginal injuries associated with pelvic fracture - a spectrum of clinical presentation and management. J Pediatr Urol 2020;16:470.e1-470.e6. 10.1016/j.jpurol.2020.05.145 32536568

[16] Tsvieli O Sergienko R Sheiner E: Risk factors and perinatal outcome of pregnancies complicated with cephalopelvic disproportion: A population-based study. Arch Gynecol Obstet 2012;285:931-936. 21932085 10.1007/s00404-011-2086-4

[17] Nielsen TF Hökegård KH: Postoperative cesarean section morbidity: A prospective study. Am J Obstet Gynecol 1983;146:911-916. 6881224 10.1016/0002-9378(83)90963-8

[18] Clayton AH Valladares Juarez EM: Female sexual dysfunction. Med Clin North America 2019;103:681-698. 10.1016/j.mcna.2019.02.008 31078200

[19] Kingsberg SA: Taking a sexual history. Obstet Gynecol Clin North America 2006;33:535-547. 10.1016/j.ogc.2006.09.002 17116499

[20] Bachmann GA Leiblum SR Grill J: Brief sexual inquiry in gynecologic practice. Obstet Gynecol 1989;73:425-427. 2915866

[21] Shulman BS Taormina DP Patsalos-Fox B Davidovitch RI Karia RJ Egol KA: Sexual function is impaired after common orthopaedic nonpelvic trauma. J Orthop Trauma 2015;29:e487-e492. 26197158 10.1097/BOT.0000000000000397

[22] Carter CT Schafer N: Incidence of urethral disruption in females with traumatic pelvic fractures. Am J Emerg Med 1993;11:218-220. 8489661 10.1016/0735-6757(93)90128-x

[23] Ter-Grigorian AA Kasyan GR Pushkar DY: Urogenital disorders after pelvic ring injuries. Cent Eur J Urol 2013;66:352-356. 10.5173/ceju.2013.03.art28 PMC3974475 24707384

[24] Curtis LA Dolan TS Cespedes RD: Acute urinary retention and urinary incontinence. Emerg Med Clin North America 2001;19:591-620. 10.1016/s0733-8627(05)70205-4 11554277

