# Supplementary material for: Association of Pelvic Trauma With Rates of Cesarean Section, Sexual Dysfunction, and Genitourinary Dysfunction in a National Database
Source: J Am Acad Orthop Surg Glob Res Rev. 2023 Apr 10;7(4):e22.00166. doi: 10.5435/JAAOSGlobal-D-22-00166 (PMC10090791; doi:10.5435/JAAOSGlobal-D-22-00166)
Supplement: SUPPLEMENTARY MATERIAL [file jagrr-7-e22.00166-s001.docx]

**SUPPLEMENTAL DATA FILE**

**Supplemental Table 1.** International Classification of Diseases, Ninth (ICD-09) and Tenth Revision (ICD-10), codes used to define pelvic and control fractures cohorts, and the Current Procedural Terminology (CPT) and ICD-09/ICD-10 codes used to define deliveries and diagnoses of sexual and genitourinary dysfunction.

| **Type** | **Code** | **Description** |
| --- | --- | --- |
| **Pelvic fracture** | | |
| ICD-10-CM | S32.1 | Fracture of sacrum |
|  | S32.3 | Fracture of ilium |
|  | S32.5 | Fracture of pubis |
|  | S32.6 | Fracture of ischium |
|  | S32.8 | Fracture of other parts of pelvis |
|  | S32.9 | Fracture of unspecified parts of lumbosacral spine and pelvis |
| ICD-9-CM | 805.6 | Closed fracture of sacrum and coccyx without mention of spinal cord injury |
|  | 805.7 | Open fracture of sacrum and coccyx without mention of spinal cord injury |
|  | 806.6 | Closed fracture of sacrum and coccyx with spinal cord injury |
|  | 806.7 | Open fracture of sacrum and coccyx with spinal cord injury |
|  | 808.2 | Closed fracture of pubis |
|  | 808.3 | Open fracture of pubis |
|  | 808.4 | Closed fracture of other specified part of pelvis |
|  | 808.5 | Open fracture of other specified part of pelvis |
|  | 808.8 | Closed unspecified fracture of pelvis |
|  | 808.9 | Open unspecified fracture of pelvis |
| **Femur/tibia fracture** | | |
| ICD-10-CM | S72.0 | Fracture of head and neck of femur |
|  | S72.1 | Pertrochanteric fracture |
|  | S72.2 | Subtrochanteric fracture of femur |
|  | S72.3 | Fracture of shaft of femur |
|  | S72.4 | Fracture of lower end of femur |
|  | S72.8 | Other fracture of femur |
|  | S72.9 | Unspecified fracture of femur |
|  | S82.1 | Fracture of upper end of tibia |
|  | S82.2 | Fracture of shaft of tibia |
|  | S82.3 | Fracture of lower end of tibia |
| ICD-9-CM | 820 | Fracture of neck of femur |
|  | 821 | Fracture of other and unspecified parts of femur |
|  | 823 | Fracture of tibia and fibula |
| **Cesarean and vaginal deliveries** | | |
| CPT Code | 59510 | Routine obstetric care including antepartum care, cesarean delivery, and postpartum care |
|  | 59514 | Cesarean delivery only |
|  | 59515 | Cesarean delivery only; including postpartum care |
|  | 59400 | Routine obstetric care including antepartum care, vaginal delivery, and postpartum care |
|  | 59409 | Vaginal delivery only |
|  | 59410 | Vaginal delivery only; including postpartum care |
| **Sexual dysfunction diagnosis** | | |
| ICD-10-CM | F52.0 | Hypoactive sexual desire disorder |
|  | F52.1 | Sexual aversion disorder |
|  | F52.22 | Female sexual arousal disorder |
|  | F52.31 | Female orgasmic disorder |
|  | F52.5 | Vaginismus not due to a substance or known physiological condition |
|  | F52.6 | Dyspareunia not due to a substance or known physiological condition |
|  | F52.8 | Other sexual dysfunction not due to a substance or known physiological condition |
|  | F52.9 | Unspecified sexual dysfunction not due to a substance or known physiological condition |
|  | N94.1 | Dyspareunia |
|  | N94.2 | Vaginismus |
|  | N94.81 | Vulvodynia |
|  | R10.2 | Pelvic/perineal pain |
|  | R37 | Sexual dysfunction, unspecified |
|  | R68.82 | Low libido |
| ICD-9-CM | 302.70 | Psychosexual dysfunction, unspecified |
|  | 302.71 | Hypoactive sexual desire disorder |
|  | 302.72 | Psychosexual dysfunction with inhibited sexual excitement |
|  | 302.73 | Female orgasmic disorder |
|  | 302.76 | Dyspareunia, psychogenic |
|  | 302.79 | Psychosexual dysfunction with other specified psychosexual dysfunctions |
|  | 306.51 | Psychogenic vaginismus |
|  | 625.0 | Dyspareunia |
|  | 625.1 | Vaginismus |
|  | 625.7 | Vulvodynia |
|  | 799.81 | Decreased libido |
|  | V41.7 | Problems with sexual function |
| **Genitourinary dysfunction diagnosis** | | |
| ICD-10-CM | R35.1 | Nocturia |
|  | R35.0 | Frequency of micturition |
|  | N39.3 | Stress incontinence |
|  | R33.8 | Other retention of urine |
|  | R33.9 | Retention of urine, unspecified |
| ICD-9-CM | 788.43 | Nocturia |
|  | 788.41 | Urinary frequency |
|  | 625.6 | Stress incontinence, female |
|  | 788.2 | Retention of urine |

**Supplemental Table 2.** Demographics of pelvic and control fracture patients.

|  | **All Fracture**  **(n = 33,328)** | **Pelvic Fracture**  **(n = 6,174)** | **Control Fracture**  **(n = 27,154)** | ***P* Value** |
| --- | --- | --- | --- | --- |
|  |  |  |  |  |
| **Sexual dysfunction^a^, n (%)** | 3,052 (9.2) | 672 (10.9) | 2,380 (8.8) | **< 0.001** |
| **Genitourinary dysfunction^a^, n (%)** | 3,776 (11.3) | 736 (11.9) | 3,040 (11.2) | 0.105 |
| Nocturia | 229 (0.7) | 43 (0.7) | 186 (0.7) | 0.921 |
| Frequency | 2,462 (7.4) | 468 (7.6) | 1,994 (7.3) | 0.521 |
| Stress incontinence | 736 (2.2) | 131 (2.1) | 605 (2.2) | 0.608 |
| Retention | 982 (2.9) | 216 (3.5) | 766 (2.8) | **0.005** |
| **Comorbidities, n (%)** |  |  |  |  |
| Diabetes | 3,917 (11.8) | 562 (9.1) | 3,355 (12.4) | **< 0.001** |
| Tobacco | 6,286 (18.9) | 1,251 (20.3) | 5,035 (18.6) | **0.002** |
| Hypertension | 6,761 (20.3) | 1,070 (17.3) | 5,691 (21.0) | **< 0.001** |
| Obesity | 4,735 (14.2) | 650 (10.5) | 4,085 (15.0) | **< 0.001** |
| Advanced Maternal Age ^b^ | 17,792 (53.4) | 2,659 (43.1) | 15,133 (55.7) | **< 0.001** |
| **Elixhauser Comorbidity Index (ECI)** | 1.93 ± 2.39 | 1.83 ± 2.38 | 1.95 ± 2.39 | **< 0.001** |
|  |  |  |  |  |

^a^ Diagnoses within five years of injury.

^b^ Advanced maternal age is defined as age greater than 35 years.
